# Potassium inward rectifier channel subfamily J member 11 mRNA expression in glioma and its significance in predicting prognosis and chemotherapy sensitivity

**DOI:** 10.3389/fonc.2026.1949969

**Published:** 2026-10-23

**Authors:** Kaijia Zhou, Bing Zhou, Qiang Gu, Tingming Lu, Qingxian Ke, Xiaoyin Liu, Jin Xu, Xinyan Zhang

**Affiliations:** 1 Clinical Oncology School of Fujian Medical University, Fujian Cancer Hospital, Fujian Branch of Fudan University Shanghai Cancer Cente, Fuzhou, China; 2 Basic Medical College of Fujian Medical University, Fuzhou, China

**Keywords:** chemosensitivity, glioma, molecular subtyping, potassium inward rectifier channel subfamily J member 11, prognosis

## Abstract

**Background:**

This study aimed to characterize the expression profile of potassium inward rectifier channel subfamily J member 11 (KCNJ11) across diverse pathological subtypes of glioma based on clinical samples from our single-center cohort. We further explored the predictive value of KCNJ11 mRNA expression for overall prognosis and temozolomide chemosensitivity in glioma patients, and validated its clinical predictive efficiency.

**Methods:**

A total of 516 glioma patients treated at the Neuro-Oncology Surgery Department of Fujian Cancer Hospital between 2006.01and 2025.01 were enrolled as the discovery cohort. Complete clinical information including gender, age, pathological subtype, WHO histological grade, IDH mutation status, 1p/19q co-deletion status, survival outcome and KCNJ11 mRNA expression levels were retrospectively collected. Univariate and multivariate Cox proportional hazards regression models were constructed to screen independent prognostic biomarkers for glioma. Three independent public glioma datasets were applied for external validation: 603 glioma cases from The Cancer Genome Atlas (TCGA), 284 cases from the Repository for Molecular Brain Neoplasia Data (REMBRANDT), and 264 cases from GSE16011 dataset in NCBI Gene Expression Omnibus (GEO). Correlations between KCNJ11 mRNA expression, clinical molecular features and overall survival (OS) were verified across all validation cohorts.

**Results:**

In our single-center discovery cohort of 516 glioma patients hospitalized from 2006 to 2025, KCNJ11 mRNA expression was significantly correlated with pathological subtype, WHO grade, IDH mutation, 1p/19q co-deletion, MGMT promoter methylation and WHO 2021 molecular classification (all P<0.05). Patients with high KCNJ11 mRNA expression exhibited markedly longer median overall survival compared with those with low expression (P<0.05). Univariate Cox regression identified age, tumor recurrence, histopathological type, WHO grade, IDH mutation, 1p/19q co-deletion, MGMT methylation and KCNJ11 mRNA expression as OS-related risk factors (all P<0.05). Multivariate Cox regression demonstrated that age (HR = 1.015, 95%CI=1.005–1.025), tumor recurrence (HR = 2.357, 95%CI=1.833–3.031), WHO histological grade (HR = 2.256, 95%CI=1.542–3.301), IDH mutation (HR = 0.717, 95%CI=0.521–0.986), 1p/19q co-deletion (HR = 0.448, 95%CI=0.283–0.709), postoperative chemotherapy (HR = 0.588, 95%CI=0.418–0.826) and KCNJ11 mRNA expression (HR = 0.687, 95%CI=0.507–0.929) were independent prognostic determinants of OS. Consistent survival trends were observed in all three external validation cohorts (TCGA, REMBRANDT, GSE16011): high KCNJ11 expression consistently predicted prolonged median OS (all P<0.05).

**Conclusion:**

KCNJ11 mRNA expression level in glioma tissues is inversely associated with tumor malignant progression, with lower expression detected in higher-grade, more malignant gliomas. KCNJ11 mRNA expression serves as an independent protective prognostic biomarker for glioma patients. Furthermore, elevated KCNJ11 expression predicts superior temozolomide chemosensitivity, indicating potential clinical value for individualized adjuvant chemotherapy stratification.

## Introduction

Glioma represents the most prevalent primary intracranial malignant neoplasm in adult populations (1). Over the past decade, multiple molecular biomarkers with prognostic and therapeutic predictive value have been identified for glioma, including isocitrate dehydrogenase (IDH) mutation (2), combined 1p/19q chromosomal deletion (3), MGMT promoter methylation (4), TERT promoter mutation (5), EGFR amplification, TP53/PTEN somatic mutations (6), MET gene amplification (7) and ATRX mutation (8). Nevertheless, high-grade glioma retains an extremely dismal clinical prognosis; glioblastoma (GBM) confers a 5-year overall survival rate of merely 5% (1). High-throughput transcriptomic profiling has become a mainstream strategy to screen novel clinical biomarkers for prognosis stratification and targeted therapeutic development, attracting extensive translational research attention (9).

Membrane ion channels exert core regulatory functions in fundamental cellular activities (10). Potassium channels modulate critical tumor biological behaviors including cell proliferation and apoptotic signaling. Voltage-gated potassium channels, transient receptor potential channels and ABC transporters have been validated as promising diagnostic markers and therapeutic targets across multiple human malignancies (11).

KCNJ11 encodes Kir6.2 subunit, which assembles with ABCC8-encoded sulfonylurea receptor (SUR1) to form ATP-sensitive potassium (KATP) channels in pancreatic β-cells. This channel complex mediates glucose-dependent insulin secretion. Pathogenic variants in KCNJ11 or ABCC8 impair channel function and trigger congenital hyperinsulinemia (12), while chronic hyperinsulinemia and hyperinsulinemic states are thought to promote colorectal carcinogenesis (13). Our previous study confirmed that ABCC8 mRNA expression independently predicts glioma prognosis and temozolomide response (14). However, the biological function and clinical significance of KCNJ11 in glioma remain unreported to date.

In this retrospective study, we systematically analyzed transcriptomic and clinical data from 516 glioma patients receiving standardized treatment at the Neuro-Oncology Surgery Department of Fujian Cancer Hospital between 2006 and 2025. We explored the correlation between KCNJ11 mRNA expression and clinical pathological/molecular characteristics, evaluated its prognostic predictive performance, and validated our findings in three independent public glioma cohorts from China and the United States. Our research aimed to establish the translational application value of KCNJ11 as a novel prognostic and chemosensitivity biomarker for glioma.

## Materials and methods

### Clinical cohort data collection (discovery cohort)

The discovery cohort consisted of 516 consecutive patients pathologically diagnosed with glioma and treated at the Neuro-Oncology Surgery Department of Fujian Cancer Hospital from 2006 to 2025. Retrospective clinical data were extracted from electronic medical records, including gender, age at diagnosis, primary/recurrent tumor status, histological subtype, WHO 2021 histological grade, IDH mutation status, 1p/19q co-deletion status, MGMT promoter methylation, postoperative radiotherapy/chemotherapy regimens, and tumor tissue KCNJ11 mRNA expression levels.

Survival data collection: Overall survival (OS) was set as the primary survival endpoint. OS was defined as the interval from the date of initial surgical resection to the date of death from any cause. Patients who remained alive or were lost-to-follow-up were censored at their last confirmed valid follow-up contact date. Survival information was collected by reviewing inpatient/outpatient electronic medical records, routine telephone follow-up, and re-visit medical records. The final follow-up cut-off date for this cohort was January 31, 2026.

According to the 2021 WHO integrated molecular classification criteria, gliomas were categorized into four molecular subgroups: IDH-mutant and 1p/19q co-deleted oligodendroglioma, IDH-mutant astrocytoma, IDH-wildtype astrocytoma, and IDH-wildtype glioblastoma. Of note, part of patients included in this discovery cohort overlaps with the patient dataset reported in our previously published ABCC8-related glioma study (Zhou et al., 2020, Sci Rep). Nevertheless, the present manuscript addresses distinct research questions focusing on KCNJ11 rather than ABCC8, and all transcriptomic quantification, statistical analyses and interpretations in this work were performed independently. The study was approved by the Institutional Review Board of Fujian Cancer Hospital (Approval No. K2023-253-01). All patients or their legal guardians provided written informed consent before clinical specimen collection and follow-up.

### External validation datasets

Three independent public transcriptomic datasets were used for cross-cohort validation:

1. TCGA cohort: 603 glioma patients from The Cancer Genome Atlas database (http://cancergenome.nih.gov);
2. REMBRANDT cohort: 284 glioma samples from Repository for Molecular Brain Neoplasia Data (https://gliovis.bioinfo.cnio.es/);
3. GEO cohort: 264 glioma cases extracted from NCBI GEO dataset GSE16011 (https://www.ncbi.nlm.nih.gov/geo/).

Consistent clinical variables and KCNJ11 expression quantification data were extracted uniformly for subsequent survival and correlation analysis.

### Tumor tissue RNA sequencing and transcriptome quantification

All glioma tissue specimens collected from our hospital’s neurosurgical operation room were immediately preserved in liquid nitrogen after resection. Total RNA was extracted from frozen tumor tissues, and RNA integrity number (RIN) ≥6.8 was verified using Agilent 2100 Bioanalyzer to screen high-quality RNA samples for RNA-seq library construction. Illumina HiSeq 2000/2500/4000 platforms were adopted for transcriptome sequencing with read lengths of 101 bp, 125 bp and 150 bp, respectively.

Raw sequencing reads were processed with STAR 2.5.2b for genome mapping (human reference genome GENCODE v19, hg19), and RSEM 1.2.31 software was applied to quantify gene expression. RSEM implements two-step normalization: normalization against transcript length to eliminate gene-length bias, followed by normalization against total library fragment counts to correct inter-sample sequencing-depth variation. Gene expression was output as FPKM (fragments per kilobase of transcript per million mapped reads). For all downstream bioinformatic analyses, KCNJ11 mRNA expression values were log_2_-transformed. Only genes with detectable expression signals were retained for downstream bioinformatic analysis. All experimental procedures complied with standard laboratory operation guidelines as previously described (15, 16).

### Statistical analysis

All statistical analyses and visualization were performed using R software (version 4.0.1, with `survival` package for survival analysis) and SPSS 25.0. For normally distributed continuous variables, two independent sample t-test was adopted for two-group comparison, while one-way ANOVA was used for multi-group comparisons.

Survival analysis details: Kaplan–Meier method was used to estimate overall survival curves for patient subgroups. Log-rank test was applied to compare survival differences between subgroups. Censored observations (alive patients and patients lost-to-follow-up) were properly incorporated in Kaplan-Meier and Cox regression models. Univariate and multivariate Cox proportional hazards regression models were constructed to screen independent prognostic factors for overall survival. Proportional hazards assumption was informally checked based on Schoenfeld residuals.

Postoperative chemotherapy was coded as a fixed binary covariate (1 = received temozolomide-based adjuvant chemotherapy immediately after surgical resection; 0 = no postoperative adjuvant chemotherapy). In our cohort, the decision to administer postoperative temozolomide chemotherapy was established shortly after surgery for every enrolled patient, with no patients required to survive an indeterminate follow-up interval before assignment to the chemotherapy group. For this reason, chemotherapy was not modelled as a time-dependent covariate in Cox regression analyses. For the primary analysis, KCNJ11 mRNA expression was dichotomized at the median log_2_-transformed FPKM value to define high- and low-expression subgroups; this median-split approach is straightforward and widely reported in comparable glioma biomarker studies. To test the robustness of our prognostic estimates, we additionally performed a complementary sensitivity analysis by entering KCNJ11 mRNA expression as a continuous log_2_-transformed FPKM variable into the multivariate Cox proportional-hazards model. Variance inflation factor (VIF) values were calculated to assess multicollinearity among co-variables entered into multivariate Cox regression, including histopathology, WHO grade, IDH mutation status, and 1p/19q co-deletion status. Variables with VIF < 5 were retained in the final multivariate model, as no severe multicollinearity was detected in our dataset. All statistical tests were two-sided, and P<0.05 was defined as the threshold for statistical significance.

## Results

### Correlation between clinical pathological features and KCNJ11 mRNA expression in our single-center cohort (n=516)

All enrolled patients hospitalized between 2006 and 2025 were stratified into high and low KCNJ11 expression groups based on the median FPKM value of KCNJ11 mRNA. The associations between clinical parameters and KCNJ11 expression were summarized in **Table 1**.

**Table 1:** Relationship between the clinical features of glioma and KCNJ11 mRNA expression in in our single-center cohort (n=516).

Parameter	Variables	N	KCNJ11 mRNA expression				χ^2^	*P*-value
			Low	%	High	%		
Sex	Female	225	105	46.7	120	53.3	1.773	0.183
	Male	291	153	52.6	138	47.4		
Age (years)*	<43	256	117	45.7	139	54.3	3.752	0.053
	≥43	260	141	54.2	119	45.8		
Recurrent	No	301	140	46.5	161	53.5	3.516	0.061
	Yes	215	118	54.9	97	45.1		
Histopathology	Oligodendroglioma	114	11	9.6	103	90.4	124.406	0.000
	Astrocytoma	218	107	49.1	111	50.9		
	Glioblastoma	184	140	76.1	44	23.9		
WHO grade	2	134	36	26.9	98	73.1	84.612	0.000
	3	198	82	41.4	116	58.6		
	4	184	140	76.1	44	23.9		
IDH mutation	Wildtype	224	167	74.6	57	25.4	95.456	0.000
	Mutant	292	91	31.2	201	68.8		
1p/19q codel	Non-codel	401	251	62.6	150	37.4	114.143	0.000
	Codel	115	7	6.1	108	93.9		
Radiotherapy	No	108	42	38.9	66	61.1	6.745	0.009
	Yes	408	216	52.9	192	47.1		
Chemotherapy	No	124	47	37.9	77	62.1	9.554	0.002
	Yes	392	211	53.8	181	46.2		
MGMT methylation	Yes	244	113	46.3	131	53.7	5.448	0.020
	No	169	98	58.0	71	42.0		
	NA	103	47	45.6	56	54.4		
WHO 2021 integrated molecular classification	Oligodendroglioma, IDH-mutant, and 1p/19q Codeleted	109	6	5.5	103	94.5	149.835	0.000
	Astrocytoma, IDH-mutant	183	85	46.4	98	53.6		
	Astrocytoma, IDH-wildtype	77	47	61.0	30	39.0		
	Glioblastoma, IDH-wildtype	147	120	81.6	27	18.4		

*Patients were divided into two groups according to a median age of 43 years.

GBM, glioblastoma.

KCNJ11 mRNA expression exhibited no statistical correlation with patient gender (P = 0.183), age stratification (P = 0.053) or tumor recurrence status (P = 0.061). Significant expression differences were detected across histological subtypes, WHO grades, IDH mutation, 1p/19q co-deletion, postoperative radiotherapy, adjuvant chemotherapy, MGMT methylation and WHO 2021 molecular classification (all P<0.001 or P<0.05).

Briefly, KCNJ11 expression ranked as follows: oligodendroglioma > astrocytoma > glioblastoma; low-grade glioma > high-grade glioma; IDH-mutant tumors > IDH-wildtype tumors; 1p/19q co-deleted tumors > non-deleted tumors; MGMT-methylated tumors > MGMT-unmethylated tumors. Patients receiving postoperative chemoradiotherapy displayed lower KCNJ11 expression than those without adjuvant treatment. In molecular subgroups, IDH-mutant 1p/19q co-deleted oligodendroglioma showed the highest KCNJ11 expression, followed by IDH-mutant astrocytoma, IDH-wildtype astrocytoma, and IDH-wildtype glioblastoma with the lowest expression level.

### KCNJ11 expression distribution stratified by single clinical variables

No significant difference in KCNJ11 mRNA expression was observed between male and female patients (**Figure 1A**). Expression levels significantly differed across age groups, primary/recurrent tumors, MGMT methylation status, histological subtypes and WHO grades (**Figures 1B–F**). Older patients, recurrent glioma, MGMT-unmethylated tumors, astrocytoma/glioblastoma and high WHO grade were associated with reduced KCNJ11 transcription.

**Figure 1:**
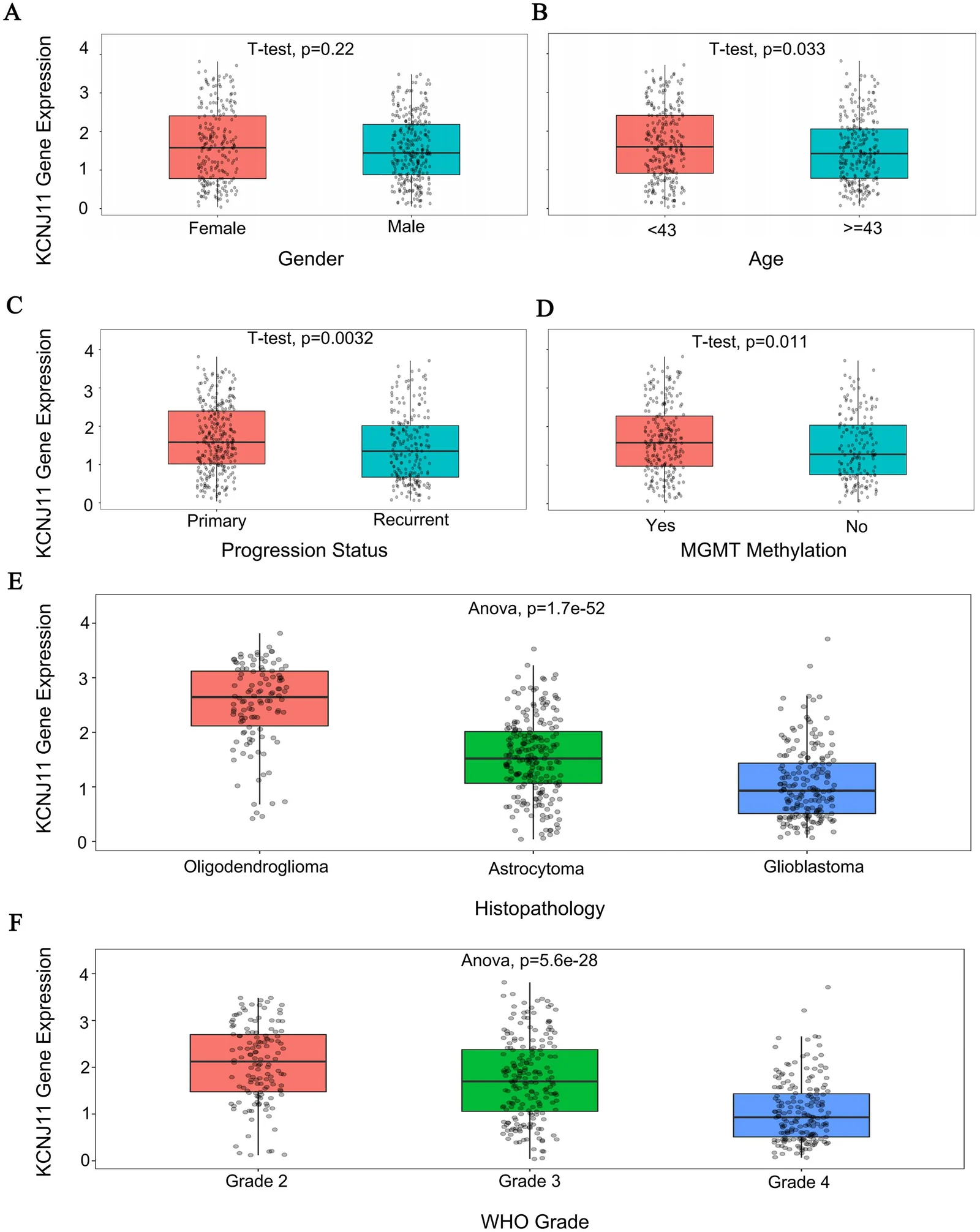
log_2_-transformed FPKM-based KCNJ11 mRNA expression stratified by gender, age, recurrence status, MGMT methylation, histological subtype and WHO grade in our single-center glioma cohort enrolled 2006–2025. **(A)** No significant expression difference between male and female patients; **(B)** Advanced age correlates with reduced KCNJ11 transcription; **(C)** Recurrent glioma displays lower KCNJ11 expression than primary tumors; **(D)** MGMT-methylated gliomas show higher KCNJ11 mRNA levels; **(E)** Expression gradient: oligodendroglioma > astrocytoma > glioblastoma; **(F)** Expression declines sequentially from WHO grade 2 to grade 4.

### KCNJ11 expression stratified by IDH mutation and 1p/19q co-deletion status

KCNJ11 mRNA expression was significantly higher in IDH-mutant gliomas versus IDH-wildtype cases, and elevated in patients carrying combined 1p/19q deletion regardless of WHO histological grade (**Figures 2A–D**).

**Figure 2:**
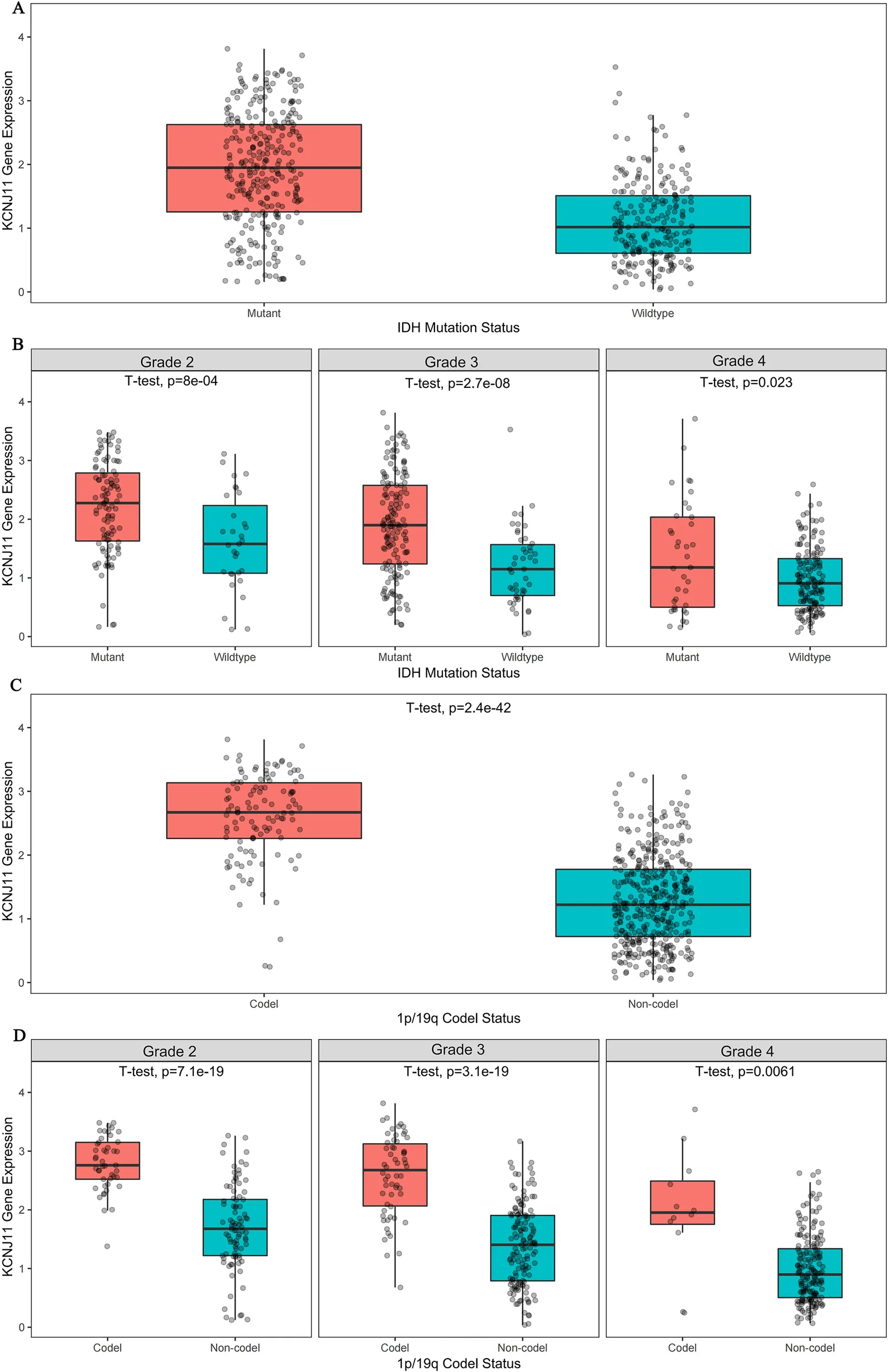
log_2_-transformed FPKM-based KCNJ11 mRNA expression stratified by IDH mutation and 1p/19q co-deletion status. **(A)** Higher expression in IDH-mutant gliomas vs IDH-wildtype; **(B)** Consistent IDH-dependent expression difference across all WHO grades; **(C)** Elevated KCNJ11 in 1p/19q co-deleted tumors; **(D)** 1p/19q co-deletion associated with higher KCNJ11 expression regardless of histological grade.

### KCNJ11 expression across four WHO 2021 molecular subgroups

Stepwise descending KCNJ11 expression was observed across four molecular subtypes: IDH-mutant 1p/19q co-deleted oligodendroglioma > IDH-mutant astrocytoma > IDH-wildtype astrocytoma > IDH-wildtype glioblastoma (**Figure 3**).

**Figure 3:**
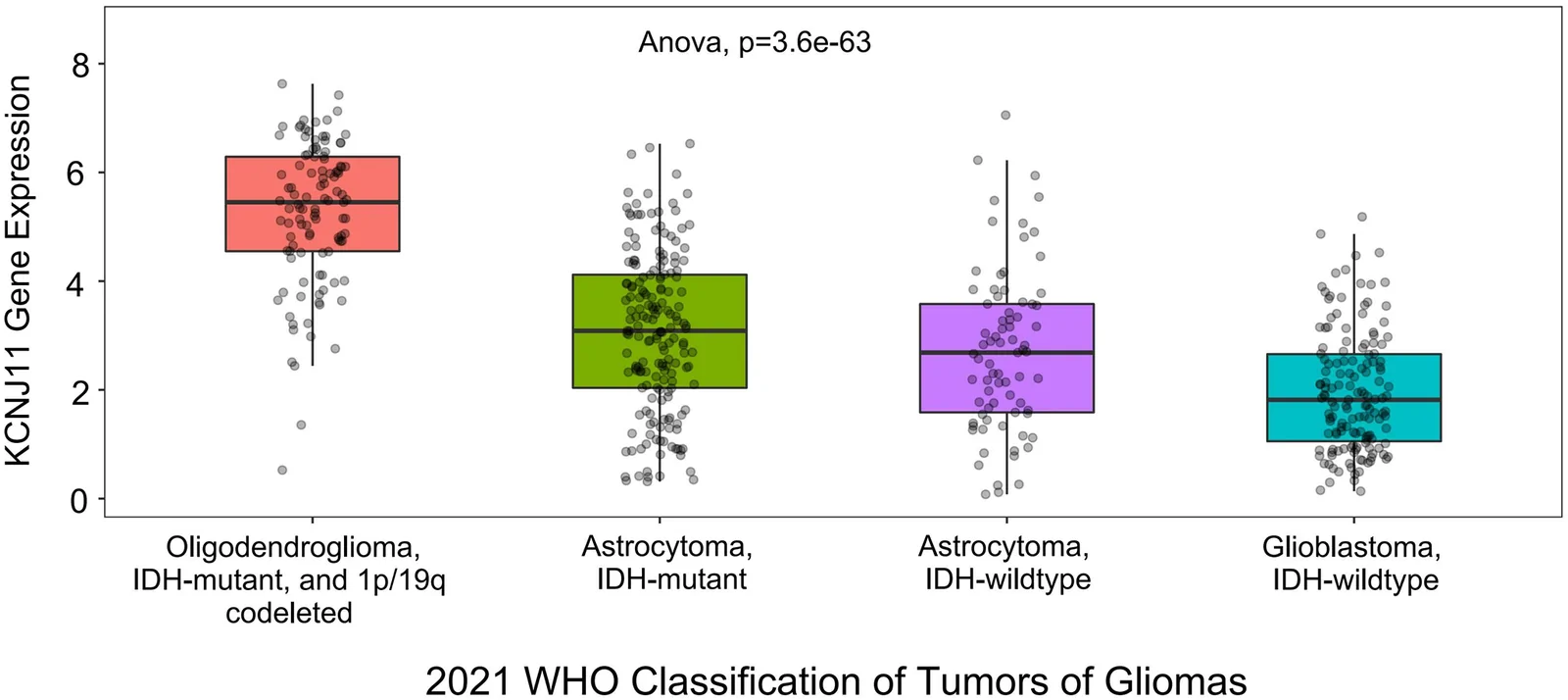
Stepwise descending log_2_-transformed FPKM-based KCNJ11 mRNA expression across four 2021 WHO integrated molecular glioma subgroups.

### Survival stratification based on KCNJ11 expression across all glioma subtypes

Kaplan–Meier survival analysis demonstrated that patients with high intratumoral KCNJ11 mRNA expression had consistently prolonged overall survival compared with low-expression counterparts across all histological and molecular glioma subgroups (**Figures 4**, **5**).

**Figure 4:**
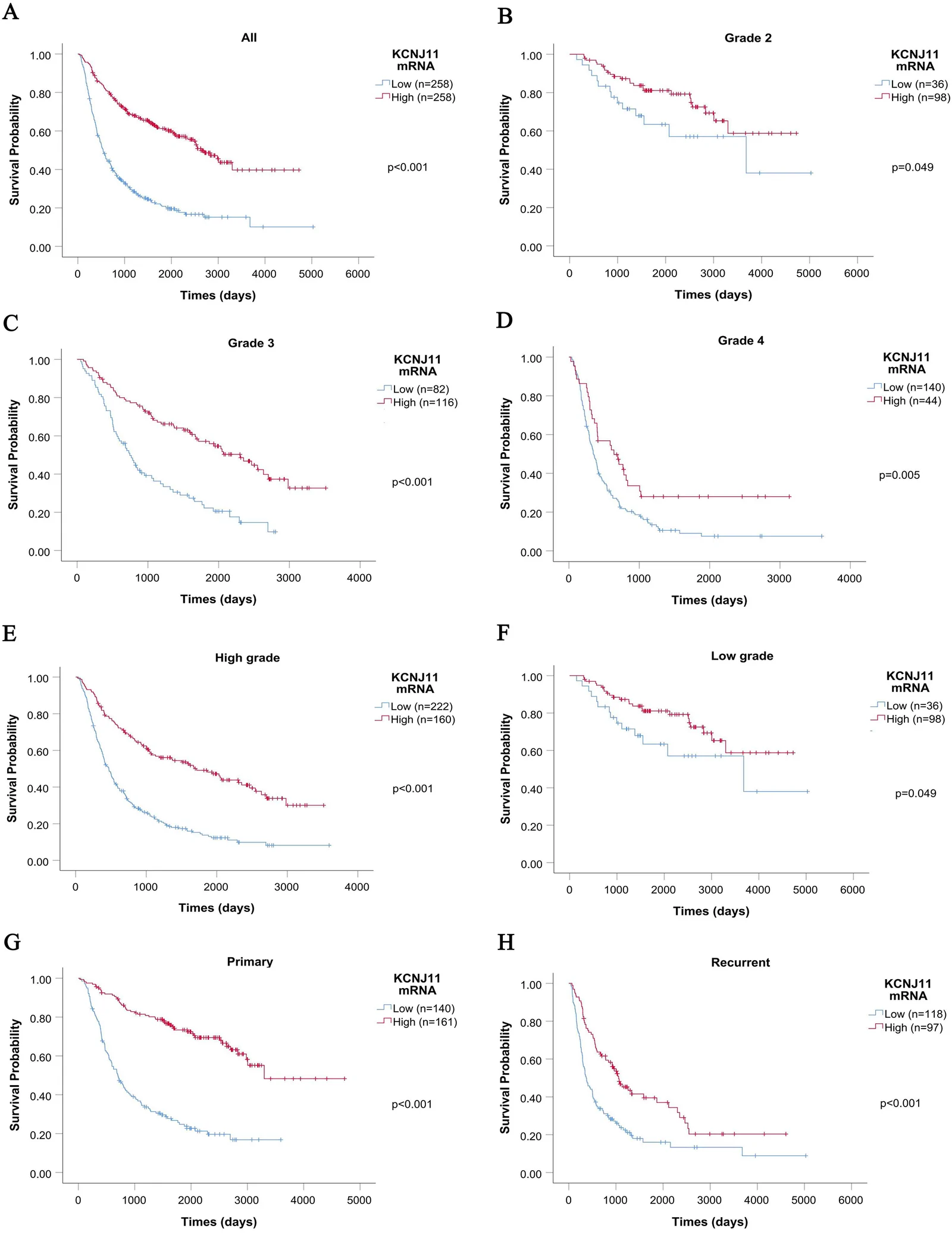
Kaplan–Meier overall survival curves stratified by KCNJ11 expression (dichotomized at median of log_2_-transformed FPKM values) across different WHO histological grades and tumor recurrence subgroups. **(A–F)** Survival stratification by histological subtypes and WHO grades; **(G, H)** Survival comparison between primary and recurrent glioma patients.

**Figure 5:**
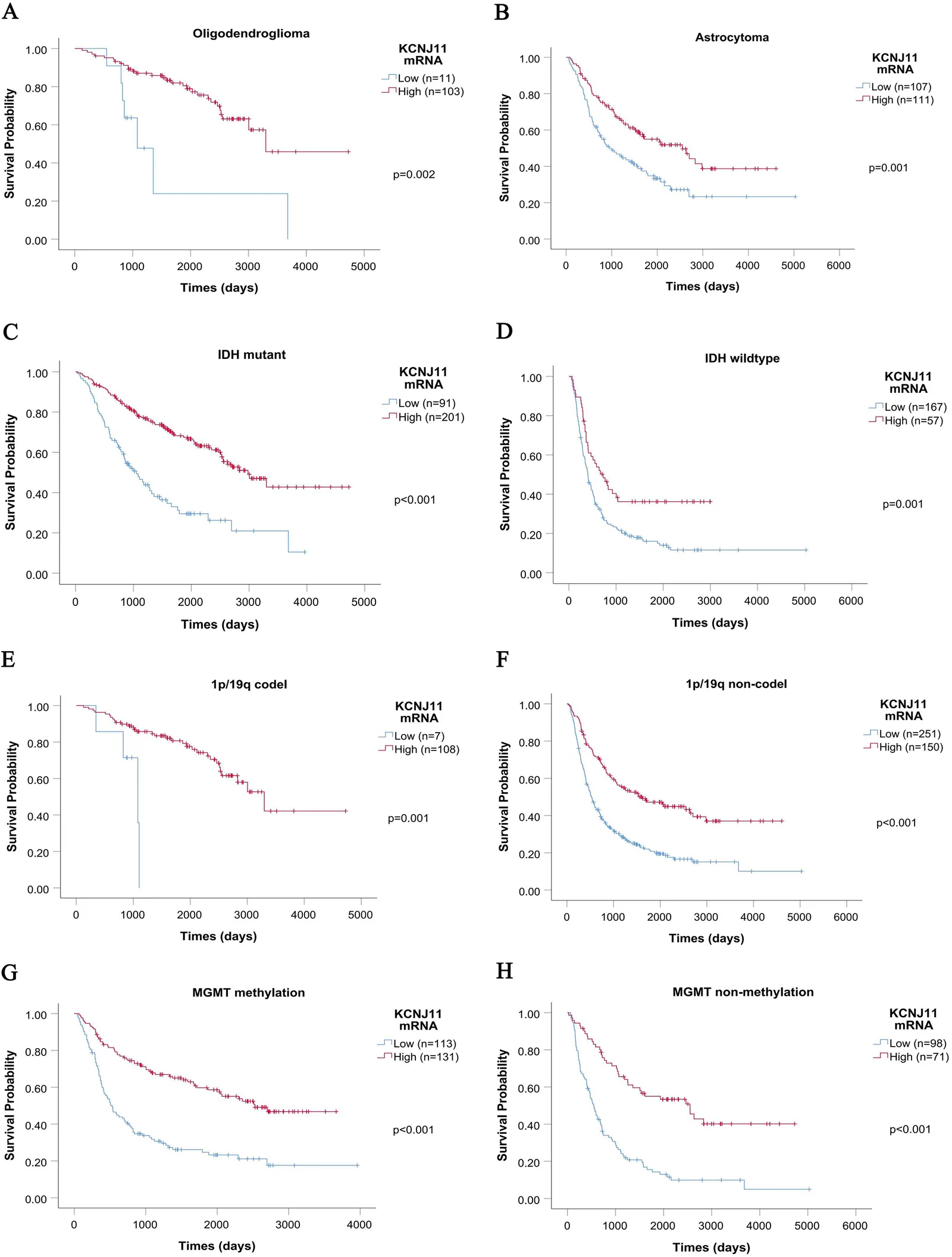
Kaplan–Meier survival curves stratified by KCNJ11 expression (dichotomized at median of log_2_-transformed FPKM values) in distinct molecular pathological subgroups. **(A, B)** Histological subtype stratification; **(C, D)** IDH mutation status stratification; **(E, F)** 1p/19q co-deletion stratification; **(G, H)** MGMT methylation stratification.

### Cox regression analysis of prognostic factors for overall survival

In complementary sensitivity analysis treating KCNJ11 mRNA expression as a continuous FPKM variable in multivariate Cox regression, KCNJ11 remained an independent prognostic factor for overall survival, consistent with our primary median-dichotomized grouping results. Variance inflation factor diagnostics for multivariate Cox covariates demonstrated all VIF values below 5, indicating the absence of severe multicollinearity among histopathology, WHO grade, IDH mutation, and 1p/19q co-deletion status in our model.

### Univariate and multivariate Cox regression in total cohort

Univariate Cox regression revealed that age, recurrence, histopathology, WHO grade, IDH mutation, 1p/19q codeletion, MGMT methylation and KCNJ11 mRNA expression were significantly associated with overall survival in glioma patients (all P<0.05). Postoperative chemotherapy was not significant in univariate analysis (P = 0.230, **Table 2**). All Variables with statistical significance in univariate analysis were subsequently entered into the multivariate Cox model without interaction term. In this baseline multivariate model, age, recurrence, WHO grade, IDH mutation, 1p/19q codeletion, chemotherapy and KCNJ11 mRNA expression emerged as independent prognostic factors for overall survival (**Table 2**).

**Table 2:** Cox analysis of factors influencing overall survival time in patients with glioma.

Variable	Univariate				Multivariate			
	*P*-value	Exp (B)	95% CI		*P*-value	Exp (B)	95% CI	
			Lower	Upper			Lower	Upper
Sex	0.447	1.091	0.871	1.366	–	–	–	–
Age	0.000	1.027	1.017	1.036	0.003	1.015	1.005	1.025
Recurrent	0.000	2.117	1.690	2.651	0.000	2.357	1.833	3.031
Histopathology	0.000	1.827	1.657	2.015	0.727	0.958	0.755	1.217
WHO grade	0.000	2.804	2.377	3.308	0.000	2.256	1.542	3.301
IDH mutant	0.000	0.321	0.256	0.403	0.041	0.717	0.521	0.986
1p/19q codel	0.000	0.282	0.199	0.400	0.001	0.448	0.283	0.709
MGMT methylation	0.046	0.782	0.614	0.996	0.484	0.915	0.714	1.173
Radiotherapy	0.467	1.109	0.839	1.465	0.488	0.882	0.620	1.257
Chemotherapy	0.230	1.176	0.903	1.533	0.002	0.588	0.418	0.826
KCNJ11 mRNA expression	0.000	0.324	0.256	0.411	0.015	0.687	0.507	0.929
KCNJ11_Chemo_inter	–	–	–		0.030	0.944	0.924	0.980

Since separate stratified significance testing between subgroups cannot serve as formal evidence of interaction, we further constructed a multivariate Cox model for the full cohort containing the KCNJ11-chemotherapy interaction term to rigorously test whether KCNJ11 expression could predict temozolomide sensitivity. The omnibus test confirmed good model performance (−2 log-likelihood = 2671.138, χ² = 234.031, df = 11, P < 0.001). After adjustment for the above confounding clinical and molecular covariates, the KCNJ11-Chemo interaction term reached statistical significance (P = 0.030, Exp(B) = 0.944, 95% CI: 0.924–0.980). This finding supported that the prognostic effect of postoperative chemotherapy on overall survival was modified by KCNJ11 mRNA expression status, providing formal statistical evidence for KCNJ11 as a potential predictive biomarker for temozolomide response. Most independent prognostic variables showed comparable hazard ratios and significance levels between models with and without the interaction term (**Table 2**).

### Stratified Cox regression by KCNJ11 expression level

Low KCNJ11 expression subgroup (**Table 3**): Postoperative temozolomide chemotherapy lost independent prognostic value; age, tumor recurrence and WHO grade remained major risk factors for poor survival. No significant OS benefit was observed in patients receiving temozolomide adjuvant therapy.

**Table 3:** Cox analysis of factors influencing overall survival in patients with glioma with low KCNJ11 mRNA expression.

Variable	Univariate				Multivariate			
	*P*-value	Exp (B)	95.0% CI		*P*-value	Exp (B)	95% CI	
			Lower	Upper			Lower	Upper
Sex	0.165	0.820	0.620	1.085				
Age	0.000	1.024	1.014	1.035	0.009	1.016	1.004	1.027
Recurrent	0.003	1.532	1.158	2.026	0.000	1.789	1.296	2.469
Histopathology	0.000	1.580	1.374	1.816	0.487	0.874	0.598	1.278
WHO grade	0.000	2.251	1.805	2.809	0.002	2.630	1.423	4.862
IDH mutant	0.000	0.493	0.364	0.668	0.257	0.793	0.532	1.183
1p/19q codel	0.264	0.568	0.211	1.533	0.734	0.834	0.294	2.370
MGMT methylation	0.222	0.829	0.613	1.120	0.557	0.911	0.667	1.244
Radiotherapy	0.102	0.737	0.512	1.062	0.121	0.682	0.421	1.107
Chemotherapy	0.727	1.067	0.743	1.531	0.186	0.732	0.461	1.162

High KCNJ11 expression subgroup (**Table 4**): Postoperative temozolomide chemotherapy remained an independent protective factor, significantly prolonging patient overall survival. Tumor recurrence, WHO grade, IDH mutation and 1p/19q co-deletion also retained independent predictive effects.

**Table 4:** Cox analysis of factors influencing survival time of patients with glioma and high KCNJ11 mRNA expression.

Variable	Univariate				Multivariate			
	*P*-value	Exp (B)	95.0% CI		*P*-value	Exp (B)	95% CI	
			Lower	Upper			Lower	Upper
Sex	0.054	1.454	0.993	2.130				
Age	0.018	1.022	1.004	1.040	0.254	1.011	0.992	1.029
Recurrent	0.000	3.219	2.202	4.706	0.000	3.874	2.523	5.948
Histopathology	0.000	1.740	1.476	2.051	0.918	0.984	0.719	1.346
WHO grade	0.000	2.578	1.963	3.387	0.005	2.059	1.239	3.422
IDH mutant	0.000	0.360	0.240	0.540	0.020	0.535	0.316	0.906
1p/19q codel	0.000	0.420	0.278	0.633	0.001	0.413	0.244	0.701
MGMT methylation	0.652	0.909	0.602	1.374	0.654	0.907	0.591	1.391
Radiotherapy	0.526	1.150	0.746	1.775	0.685	1.120	0.648	1.934
Chemotherapy	0.730	0.932	0.625	1.390	0.006	0.471	0.276	0.806

### Survival validation in three independent external cohorts

Consistent survival results were replicated in TCGA, REMBRANDT and GSE16011 validation cohorts. In all three datasets, high KCNJ11 mRNA expression was significantly correlated with longer median overall survival (Log-rank χ²=79.317, P<0.001; χ²=16.108, P<0.001; χ²=6.580, P = 0.010, respectively), as visualized in **Figures 6A–C**.

**Figure 6:**
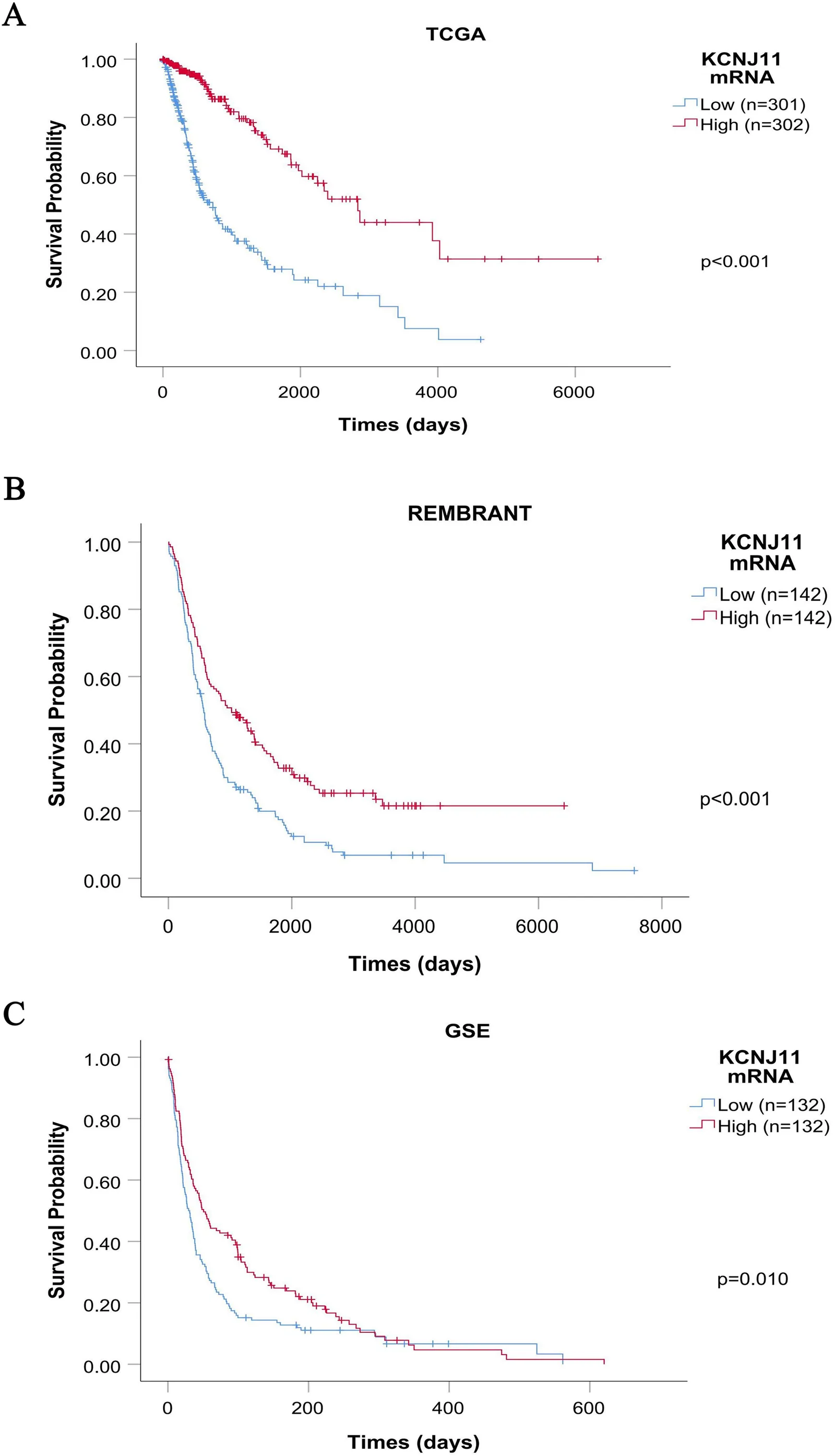
External validation of KCNJ11 survival predictive effect in three independent public glioma datasets. KCNJ11 expression subgroups were defined using median-split of normalized expression values within each respective dataset. **(A)** TCGA cohort (n=603); **(B)** REMBRANDT cohort (n=284); **(C)** GSE16011 cohort (n=264). High KCNJ11 expression consistently predicts prolonged overall survival in all validation cohorts.

## Discussion

The human KCNJ11 gene is mapped to chromosome 11p15.1, containing a single exon encoding a 390-amino-acid Kir6.2 protein. Kir6.2 assembles with ABCC8-encoded SUR1 to form the pancreatic β-cell ATP-sensitive potassium channel complex (KATP) at a 4:4 stoichiometry (17, 18). KATP channels are central mediators of glucose-stimulated insulin secretion: elevated intracellular ATP/ADP ratio induced by glucose metabolism closes KATP channels, triggering membrane depolarization, voltage-gated Ca^2+^ channel opening, calcium influx and insulin granule exocytosis. Gain- or loss-of-function KCNJ11 variants disrupt channel activity, resulting in congenital diabetes or persistent hyperinsulinemic hypoglycemia (19).

Emerging evidence links KCNJ11 dysregulation to multiple malignant tumors, though its mechanistic role in oncogenesis remains poorly characterized. Early research confirmed KCNJ11’s core function in insulin secretion; (20) epidemiological studies established a close correlation between diabetes mellitus and tumor incidence. (17) Diabetic breast cancer patients exhibit higher mortality and inferior prognosis, identifying diabetes-related KCNJ11 as a potential breast cancer prognostic biomarker. (18) Translational experiments further verified reduced KCNJ11 protein abundance in breast carcinoma tissues, with high KCNJ11 expression predicting improved clinical outcomes, partially regulated by gene methylation modification (21).

KCNJ11 single-nucleotide polymorphisms are associated with adult metabolic syndrome, including central obesity, hypertension, hyperglycemia and dyslipidemia. Metabolic syndrome induces insulin resistance and compensatory hyperinsulinemia, which directly accelerates proliferation of multiple epithelial malignancies such as colorectal and breast cancer. (19) Body weight and obesity also modulate KCNJ11 transcriptional levels, providing a potential mechanistic link between metabolic disorders and tumor progression. (22) In hepatocellular carcinoma research, KCNJ11 potassium channel mRNA expression declines during cirrhosis-to-HCC malignant transformation, consistent with a tumor-suppressive expression pattern (23).

Prior to this work, no research has reported KCNJ11 expression and clinical relevance in glioma. Our single-center retrospective cohort of 516 glioma patients treated at Fujian Cancer Hospital between 2006 and 2025 demonstrated that KCNJ11 mRNA expression inversely correlates with tumor malignant degree. Higher KCNJ11 levels were detected in primary, low-grade, IDH-mutant, 1p/19q co-deleted oligodendroglioma, while recurrent, high-grade, IDH-wildtype glioblastoma exhibited markedly suppressed KCNJ11 transcription. These findings support KCNJ11 as a molecular marker reflecting glioma malignant progression.

Kaplan–Meier survival curves across all histological and molecular subgroups confirmed that high KCNJ11 expression correlates with prolonged overall survival. Multivariate Cox regression further validated KCNJ11 mRNA expression as an independent protective prognostic biomarker, independent of traditional clinical and molecular risk factors. Most importantly, stratified Cox regression revealed a novel predictive function of KCNJ11 for temozolomide chemosensitivity: patients with high KCNJ11 expression derive significant OS benefit from postoperative temozolomide chemotherapy, whereas low-KCNJ11 patients show no survival improvement with standard temozolomide adjuvant treatment. This indicates KCNJ11 could guide individualized chemotherapy decision-making for glioma patients. These interaction-model results complement our stratified subgroup observations and avoid the statistical limitation of inferring interaction solely by comparing significance across separate subgroups. Our data suggested that KCNJ11 expression may modulate therapeutic benefit from temozolomide chemotherapy in glioma patients, which merits further external validation before clinical translation.

Several limitations should be acknowledged in this study. First, our analysis solely relies on transcriptomic mRNA expression data; further cellular, animal and tissue protein validation is required to elucidate the upstream transcriptional regulatory network and downstream tumor-suppressive signaling pathways mediated by KCNJ11 in glioma. Second, although three independent public cohorts (TCGA, REMBRANDT, and GSE16011) externally validated the prognostic association between KCNJ11 mRNA expression and overall survival, these public datasets lack complete standardized postoperative chemotherapy information, so we could not replicate the formal KCNJ11-chemotherapy interaction test in external cohorts. Our conclusion regarding KCNJ11 as a predictive biomarker for temozolomide chemosensitivity is derived exclusively from our single-center retrospective discovery cohort. Multi-center prospective cohorts with complete chemotherapy treatment records are needed for further external validation of this predictive interaction effect before clinical translation can be considered. Third, postoperative chemotherapy was entered into Cox regression as a fixed binary covariate rather than a time-dependent variable. Even though chemotherapy assignment was determined shortly after surgery with no prolonged immortal-time window in our cohort, theoretical immortal-time bias cannot be completely excluded in this retrospective observational study. Future validation analyses adopting time-dependent Cox models would help to further confirm our hazard-ratio estimates. Fourth, our primary analysis dichotomized KCNJ11 mRNA expression at median FPKM, which represents an arbitrary cut-off. Although sensitivity analysis treating KCNJ11 as a continuous variable supported our main findings, median-split grouping may result in loss of quantitative information. In addition, several strongly inter-correlated clinical-molecular variables were simultaneously included in multivariate Cox regression; despite acceptable VIF values excluding severe multicollinearity, mild correlation between these covariates cannot be fully ruled out.

## Conclusion

KCNJ11 mRNA expression in glioma tissues is negatively correlated with tumor malignant grade, with lower transcription detected in more aggressive glioma subtypes. KCNJ11 mRNA level acts as an independent favorable prognostic biomarker for glioma patients. Critically, elevated KCNJ11 expression predicts superior temozolomide chemosensitivity, providing a novel molecular indicator to stratify glioma patients for personalized alkylating-agent chemotherapy.

## Data Availability

The datasets presented in this study can be found in online repositories. The names of the repository/repositories and accession number(s) can be found in the article/supplementary material.
